# *Codonopsis pilosula* Polysaccharide Improved Spleen Deficiency in Mice by Modulating Gut Microbiota and Energy Related Metabolisms

**DOI:** 10.3389/fphar.2022.862763

**Published:** 2022-04-26

**Authors:** Lingya Cao, Changli Du, Xiaolu Zhai, Jiankuan Li, Jingyi Meng, Yunyun Shao, Jianping Gao

**Affiliations:** School of Pharmaceutical Science, Shanxi Medical University, Taiyuan, China

**Keywords:** *Codonopsis pilosula* polysaccharide, spleen deficiency syndrome, 16S rRNA gene sequencing, targeted metabolomics, *Lactobacillus*

## Abstract

Codonopsis Radix (CR) is an important traditional Chinese medicine used for the treatment of spleen deficiency syndrome (SDS). *Codonopsis pilosula* polysaccharides (CPP) in CR are considered to be responsible for tonifying the spleen function; however, the mechanisms of the polysaccharides have remained unclear. This study aimed to investigate the treatment mechanisms of CPP in SDS mice using a combinational strategy of 16S rRNA gene sequencing and targeted metabolomics. Here, studies demonstrated that CPP had invigorating effect *in vivo* in Sennae Folium-induced SDS in mice by organ indexes, *D*-xylose determination, gastrointestinal hormones levels and goblet cells observation. Antibiotic treatment revealed that the intestinal microbiota was required for the invigorating spleen effect of CPP. Furthermore, gut microbiota analysis found that CPP significantly enriched probiotic *Lactobacillus* and decreased the abundance of some opportunistic pathogens, such as *Enterococcus* and *Shigella*. The metabolic profile analysis of the colonic content revealed that 25 chemicals were altered significantly by CPP, including amino acids, organic acids, fatty acids, carbohydrates and carnitine etc., which are mainly related to “energy conversion” related processes such as amino acids metabolism, tricarboxylic acid cycle, and nitrogen metabolism. Spearman’s correlation assays displayed there were strong correlations among biochemical indicators-gut microbiota-metabolomics. In summary, these results provided a new perspective for CPP improving SDS by regulating energy metabolism related bacteria and pathways.

## Introduction

In traditional Chinese medicine (TCM), the term spleen does not refer to the spleen organ defined in modern medicine. Instead, “spleen govern transportation and transformation” is regarded as the primary function of TCM spleen. In other words, spleen plays the leading role in the process of food digestion, absorption and transportation. Modern research has confirmed that it is closely related to the gastrointestinal systems, as well as the nervous, endocrine, blood circulation and immune systems (Wu, 1998; Tu et al., 2020). Spleen deficiency syndrome (SDS) is a common syndrome of TCM that is associated with digestive system diseases (Ning et al., 2021), gastrointestinal hormone disorders and hypoimmunity (Sun et al., 2016; Wang et al., 2020). Clinically, SDS is characterized by dyspepsia, diarrhea, chills, lazy words, shortness of breath and fatigue (Wang N. et al., 2020). Several studies have reported that SDS is closely related to energy metabolism disorders (Zheng et al., 2014), and that the function of mitochondria is abnormal (Liu et al., 2021). Furthermore, SDS can aggravate the pathogenesis of various diseases, such as chronic atrophic gastritis (Zhang et al., 2014), irritable bowel syndrome (Wang et al., 2020), and Alzheimer’s disease (Yu et al., 2014). Thus, invigorating the spleen is an effective strategy to prevent and treat diseases.

Codonopsis Radix (CR), also known as “Dangshen” in Chinese, is derived from the roots of *Codonopsis pilosula* (Franch.) Nannf, *C. pilosula* Nannf. var. *modesta* (Nannf.) L. D. Shen, and *C. tangshen* Oliv. CR is widely used to invigorate the spleen and tonify the lung in the clinical practice of TCM. CR was used as a “monarch drug” in several famous treatments of TCM, such as Shenling Baizhu power and Zhipixie prescription, and has a good curative effect in treating diarrhea due to SDS in children (Jin, 2020). Chemical studies have found that the main active constituents of CR are polysaccharides, lignans, and polyacetylenes (Gao et al., 2018; Luan et al., 2021). Among them, *Codonopsis pilosula* polysaccharide (CPP) has attracted considerable attention owing to its immunomodulatory, antioxidant, anti-tumor, anti-fatigue and prebiotic activities (Zou et al., 2020).

Due to the carbohydrate active enzymes are not encoded in human genome, but exit in the genes of bacterium (Huang et al., 2017). Most plant polysaccharides rely on gut microbiota to degrade into various small substances available to the host that includes, but not limited to, short chain fatty acids (SCFAs). Among these, butyrate can act as energy resource for colonocyte directly while acetate can be used as a substrate to synthesize cholesterol and long-chain fatty acids in the liver (Liu et al., 2022). Meanwhile, polysaccharide treatment benefited the intestinal bacterial community mainly accompanied by the alteration of amino acid metabolism, energy metabolism, and tricarboxylic acid (TCA) metabolism (Wan et al., 2020; Li et al., 2021). In patients with SDS and experimental animals, the composition of the gut microbiota and metabolic profile are significantly different from those of healthy individuals (Zheng et al., 2014; Lin et al., 2018b; Ma et al., 2019). In a previous study, we demonstrated that the polysaccharides are mainly metabolized in the colon and used by probiotics (Li et al., 2018), however, the specific role of CPP in the regulation of the intestinal microbiota in SDS mice remained unclear.

Recent studies have indicated that intestinal bacteria contribute to the human metabolic phenotype by producing bioactive metabolites and transporting them to the host system (Agus et al., 2021). Metabolomic analyses can measure a broad range of metabolites in tissues or biofluids and are consistent with the holistic view of TCM (Guo et al., 2012). Therefore, the integration of gut microbiota and metabolomics is a new strategy for studying the effectiveness evaluation and mechanisms of TCM in the treatment of diseases (Qiu et al., 2014; Zhang and Zhao, 2016).

In the present study, the invigorating spleen effects of CPP in SDS mice were investigated. An *in vivo* treatment with antibiotics was performed to confirm whether CPP exert effects in a gut microbiota dependent manner. We aimed to explore the related mechanisms of CPP acted on SDS *via* combing 16S rRNA gene sequencing techniques and targeted metabolomics. Specifically, we employed ultra-high-performance liquid chromatography-quadrupole time-of-flight tandem mass spectrometry (UPLC-Q-TOF-MS) for the targeted metabolomics analysis. Furthermore, the relationship between the gut microbiota and the metabolites involved in the invigorating spleen effect of CPP was determined with a correlation analysis.

## Materials and Methods

### *Codonopsis pilosula* Polysaccharide Extraction

The dried roots of *Codonopsis pilosula* (Franch.) Nannf. was obtained from Pingshun County, Shanxi Province, China (1,460 m altitude, 36°14′N, 113°30′E) in 2018 after 2 years of growth, and identified by Professor Jianping Gao (Shanxi Medical University). The roots (3.0 kg) were dissected into segments, soaked with 9 L water overnight and extracted by refluxing method at 100°C for 2 h. The residues were then successively extracted with 5 L water for 1 h, and with 4 L water for 1 h in the same way, respectively. The extracts were filtered, combined and centrifuged at 1,160 × g for 15 min. The extract recovered after centrifugation was concentrated under vacuum to a quarter of its volume. Ethanol (95%) was added to 80% ethanol concentration, after stored at 4°C overnight. The precipitate was collected by centrifugation and was washed three times with ethanol, acetone and petroleum ether successively to obtain CPP (495 g) (Zhang et al., 2020). The total carbohydrate content of CPP was determined by the phenol sulfuric acid method with glucose as standard samples (Zhen et al., 2014). High-performance gel permeation chromatography (HPGPC) was performed to analyze CPP using an Agilent 1260 HPLC system equipped with TSK gel G4000PWXL column (7.8 mm × 30 cm, 10 μm) and RID-G1362A detector (Santa Clara, CA, United States). A volume of 20 μl CPP (1 mg/ml) was injected to the system and eluted with water at a flow rate of 0.3 ml/min with the column temperature maintained at 35°C.

### Preparation of Sennae Folium Solutions

*Cassia angustifolia* Vahl (Batch No. 20171201) was provided by the Shanxi Traditional Chinese Medicine Institute and was identified by Professor Jianping Gao (Shanxi Medical University). The dried leaves of *C. angustifolia* Vahl. (5.0 kg) underwent twice reflux extraction with 50 L water for 30 min. The solvent was collected, concentrated to 2 g/ml and stored at −80°C.

### Animal Experiments

Thirty male ICR mice [8 weeks old, 18 ± 2 g, animal license No. SCXK (Jing) 2019-0010, SPF grade] were purchased from SPF Biotechnology Co., Ltd. (Beijing, China). Mice were housed under pathogen-free conditions and allowed to consume food and sterile water ad libitum in a temperature-controlled room (22 ± 2°C). After 1 week of adaptive feeding, mice were divided into three groups (*n* = 10 mice/group) using the random number table method: control group, SDS group and SDS + CPP group. Mice in the control group were administered 0.1 ml 0.9% saline solution per 10 g of body weight *via* gavage for 11 weeks once daily. Mice in the SDS group were treated with Sennae Folium (20 g/kg body weight) for 11 weeks once daily. Mice in the SDS + CPP group received Sennae Folium doses as mice in SDS group for the first 5 weeks; thereafter, mice simultaneously received Sennae Folium and CPP solution (1600 mg/kg) once daily for 6 weeks more. The dosage of CPP was determined by previous experiments (Meng et al., 2021). At the end of the experiment, the mice were anesthetized with isoflurane, blood were sampled from the mouse orbit before they were sacrificed by spinal dislocation. This study was approved by the Animal Ethics Committee of Shanxi Medical University (No. 2017001). All of the animal experiments were performed in accordance with the National Administration Regulations on Laboratory Animals issued by the State Committee of Science and Technology of China. This experimental protocol complied with the Animal Research: Reporting of *in vivo* Experiments (ARRIVE) Guidelines 2.0 (Percie du Sert et al., 2020).

### Pyrogallol Method and Enzyme-Linked Immunosorbent Assays

Mice were administered with 2% *D* -xylose solution (10 ml/kg body weight) *via* gavage 1 h before blood collection. The samples were centrifuged at 650 × g for 10 min at 4°C to separate the serum. The blood levels of *d* -xylose were determined by the pyrogallol method using *D* -xylose assay kit (Nangjing Jiancheng Bioengineering Institute, Nanjing, China) and an ultraviolet-visible spectrophotometer (Shanghai YoKe Instrument Co., Ltd., Shanghai, China) at 554 nm. The serum levels of gastrin (GAS), somatostatin (SS) were determined using GAS and SS ELISA kits purchased from Elabscience Biotechnology Co., Ltd. (Lot No JROFHBQUGW and PT8BPNF3SJ, Wuhan, China) according to the manufacturer’s instructions, and measured at a wavelength of 450 nm. Amylase (AMS) was determined using AMS ELISA kit obtained from Shanghai MLBIO Biotechnology Co. Ltd. (Shanghai, China) following the manufacturer’s instructions.

### Antibiotic Treatment

Twenty male mice were randomly distributed into two groups: Ab group and Ab + CPP group. Mice in Ab group received the treatment of SDS group, while mice in Ab + CPP group received the treatment of SDS + CPP group. During the last 2 weeks of modeling, each mouse was administered 0.2 ml of an antibiotic mixture (intragastric administration) twice daily. The mixture antibiotic solution of penicillin (10 mg/ml), metronidazole (10 mg/ml), vancomycin hydrochloride (5 mg/ml), neomycin sulfate (10 mg/ml), and amphotericin B (0.1 mg/ml) dissolved in sterile distilled water (Cao et al., 2017). During the treatment, antibiotics (Streptomycin 200 mg/L, gentamicin 170 mg/L, ciprofloxacin 144 mg/L and bacitracin 200 mg/L) were applied to the drinking water of Ab and Ab + CPP groups. The drinking water was changed once every 2 days (Liu et al., 2019).

### 16S rRNA Gene Sequencing Analysis

Genomic DNA was extracted from colonic contents of control group, SDS group and SDS + CPP group (*n* = 6) using TIANamp Stool DNA kit (Tiangen Biotech Co. Ltd., Beijing, China). DNA quality and quantity were assessed using Nanodrop (Thermo Fisher Scientific Inc., Waltham, United States) and 1.2% agarose gel electrophoresis. The V3-V4 regions of the16S rRNA gene were amplified by PCR using the universal primers (forward: 5′-ACT​CCT​ACG​GGA​GGC​AGC​A-3′ and reverse: 5′-GGACTACHVGGGTWTCTAAT-3′). The TruSeq^®^ Nano DNA LT Library Prep kit of Illumina (San Diego, CA, United States) was used to prepare the sequencing library. The quality-qualified library was paired-end sequenced using NovaSeq (Illumina, San Diego, United States) at Shanghai Personalbio Biotechnology Co., Ltd., Shanghai, China.

### Colonic Content Metabolomics

After CPP treatment, colons were opened longitudinally and the colonic contents from control group, SDS group and SDS + CPP group (*n* = 6) were collected into a sterile tube, all of the colon contents were immediately frozen in liquid nitrogen and next stored at −80°C for metabolic profiling analyses. Approximately 5 mg of each lyophilized colonic content was weighed, 25 μl of water and 120 μl of a methanol solution containing internal standard were added, and each sample was homogenized with zirconium oxide beads to extract the metabolites. The supernatant (20 μl) was transferred to a 96-well plate and centrifuged at 18,000 × g for 20 min. Freshly prepared derivative reagents (20 μl) were then added to each well. The plate was sealed and the derivatization was carried out at 30°C for 60 min. After derivatization, 330 μl of ice-cold 50% methanol solution was added to dilute the sample. The plate was stored at −20°C for 20 min followed by 4,000 × g centrifugation at 4°C for 30 min. The supernatant (135 μl) was transferred to a new 96-well plate with 10 μl internal standards in each well. Serial dilutions of the derivatized stock standards were added to the left wells. The plate was then sealed for UPLC-Q-TOF-MS analysis.

The samples were analyzed using UPLC-Q-TOF-MS (ACQUITY UPLC-Xevo TQ-S, Waters Corp, Milford, MA, United States). All samples were injected (5 μl) into an ACQUITY UPLC BEH C18 column (2.1 × 100 mm, 1.7 μm) at a flow rate of 0.4 ml/min. The mobile phase A was 0.1% formic acid in water, and B was acetonitrile-isopropyl alcohol (70:30, v/v). The chromatographic separation was conducted by the following gradient elution program: 0–1 min, 5% B; 1–11 min, 5%–78% B; 11–13.5 min, 78%–95% B; 13.5–14 min, 95%–100% B; 14–16 min, 100% B; 16–16.1 min, 100%–5% B; and 16.1–18 min, 5% B. The column temperature was set to 40°C (Xie et al., 2021).

### Statistical Analysis

Microbiome data were performed using QIIME 2 2019.4 (Bolyen et al., 2018). After raw data were demultiplexed using the demux plugin, the cutadapt plugin was called to excise the primer fragment of the sequence (Martin, 2011). And then Divisive Amplicon Denoising Algorithm 2 (DADA2) pipeline was employed to turn sequences into denoised, merged, chimera-free Amplicon Sequence Variants (ASVs) (Callahan et al., 2016). Non-singleton ASVs were aligned with mafft (Katoh et al., 2002) and used to construct a phylogeny with fasttree2 (Price et al., 2009). Then, ASVs were annotated using a trained Naïve Bayes classifier (Bokulich et al., 2018). For alpha diversity, the indexes of Simpson, Shannon, Chao 1 and Paith_pd were calculated by QIIME2. Principal component analysis (PCA) quantifies the degree of difference in species composition between samples with R package (v3.2.0) (Ramette, 2007). Cluster analysis was carried out using the unweighted pair group method with arithmetical means (UPGMA) (Ramette, 2007). Linear discriminant analysis effect size (LEfSe) was used for biomarker discovery among the different groups, with a linear discriminant analysis (LDA) score >4 and statistical significance set at *p* < 0.05 (Segata et al., 2011).

Metabolomic data matrix was imported into SIMCA software for the principal component analysis (PCA), which can visualize the trends and outliers among the groups. Partial least squares-discriminant analysis (PLS-DA) was used to compare the differences between the groups. S-plots and variable importance in projection (VIP) >1 were considered significantly different between groups. The online sources of HMDB (http://www.hmdb.ca), KEGG (http://www.genome.jp/kegg/), and METLIN (http://www.metlin.scipps.edu/) were used for data processing. The screened differential metabolites were analyzed using MetaboAnalyst (http://www.metaboanalyst.ca/) for enrichment analysis and pathway analysis. We used the Spearman’s correlation analyses to determine the correlation between the altered colonic content compounds and bacterial profiles or biochemical parameters; *p* < 0.05 was considered as statistically significant.

Data analyzed by One-way ANOVA and Student’s t-test were first assessed for normality test. As data not normally distributed, nonparametric Kruskal Wallis test were performed with the SPSS software (version 17.0; *p* < 0.05).

## Results

### Characterization of *Codonopsis pilosula* Polysaccharide by High-Performance Gel Permeation Chromatography

According to the linear regression equation of glucose: Y = 0.0162X + 0.0185 (R^2^ = 0.9996), the CPP contains 81.3% of total carbohydrate. HPGPC revealed that CPP was mainly composed of polysaccharides with average molecular weights of 3.25 × 10^6^–5.74 × 10^5^ Da (17.388–21.394 min), 3.78 × 10^3^ Da (33.449 min), and 1.59 × 10^3^ Da (35.102 min) (Supplementary Figure S1). The polysaccharides of 3.78 × 10^3^ Da and 1.59 × 10^3^ Da were the main components in CPP because the peak area at 33.449 and 35.102 min accounted for more than 90% of total area. Meanwhile, the ^13^C-NMR spectrum of CPP exhibited clear signals of inulin-type fructans (Supplementary Figure S2) (Li J. et al., 2017).

### Monosaccharide Composition

As shown in Supplementary Table S1, CPP was composed of six monosaccharides: fructose, glucose, arabinose, galactose, glucosamine hydrochloride, and galactosamine hydrochloride. The content of fructose was the highest (with a mole ratio of 61.8), and galactosamine hydrochloride was the lowest (with a mole ratio of 0.2).

### *Codonopsis pilosula* Polysaccharide Treatment Ameliorates Spleen Deficiency Syndrome

**FIGURE 1:**
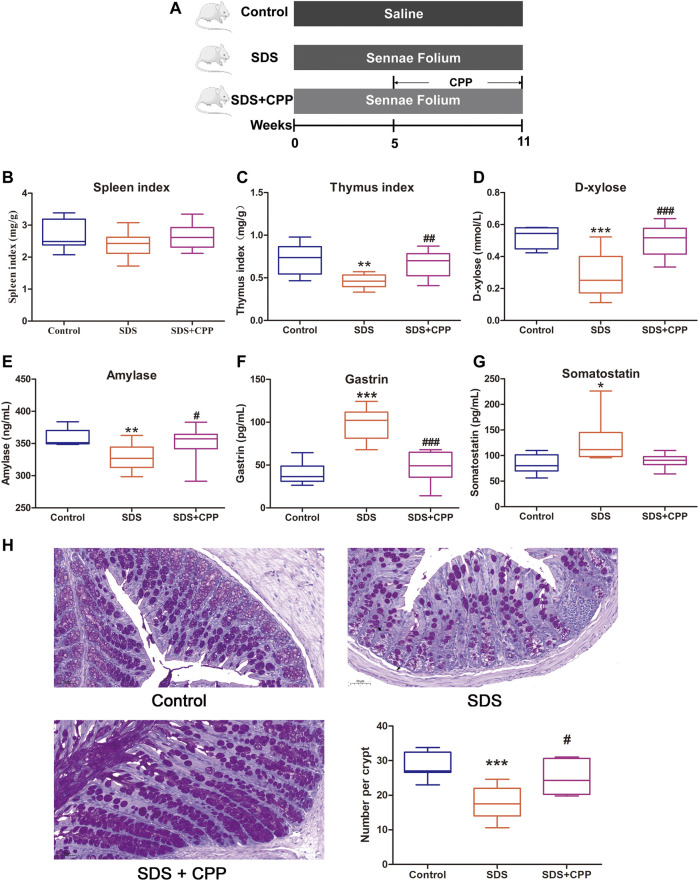
CPP mitigated Sennae Folium-induced SDS. Boxplots provide range, mean, minimum and maximum value. **(A)** Timeline of experimental process of mice in each group. **(B)** Effect of CPP on spleen index. **(C)** Changes in thymus index. **(D)** Serum concentration of *d* -xylose. **(E)** Serum levels of amylase reflecting digestive ability. **(F)** Serum concentration of GAS. **(G)** Serum concentration of SS. **(H)** Representative PAS-stained colonic sections (×200 magnification) in each group; and number of goblet cells per crypt in each group. Data were expressed as mean ± SEM (*n* = 8–10). *p < 0.05, **p < 0.01, ***p < 0.001 *vs.* control group; ^#^ *p* < 0.05, ^##^ *p* < 0.01, ^###^ *p* < 0.001 *vs.* SDS group. SDS: spleen deficiency syndrome group. SDS + CPP: spleen deficiency syndrome + *Codonopsis pilosula* polysaccharide treatment group.

The protective effect of CPP against SDS was evaluated in a Sennae Folium-induced mouse model (Figure 1A). As shown in Figure 1B, there was no significant difference in the spleen index among the control group, the SDS group and the SDS + CPP group After 6 weeks of administration of Sennae Folium, the thymus index, *D* -xylose absorption, and AMS were significantly lower in the SDS group than in the control group (*p* < 0.05) (Figures 1C–E). Moreover, the gastrointestinal hormones such as GAS and SS significantly increased in the SDS group compared to the control group (*p* < 0.05) (Figures 1F,G). And goblet cell loss was clearly observed in colonic sections of the SDS group (Figure 1H). However, SDS + CPP group presented markedly elevated levels of thymus index, serum *D* -xylose, AMS and goblet cells (*p* < 0.05) (Figures 1C–E,H) and reversed the elevated levels of gastrointestinal hormones (*p* < 0.05) (Figures 1F,G). These results demonstrate that CPP has the potential to alleviate the Sennae Folium-induced SDS in mice.

### *Codonopsis pilosula* Polysaccharide Ameliorates Gut Microbial Dysbiosis in Spleen Deficiency Syndrome Mice

**FIGURE 2:**
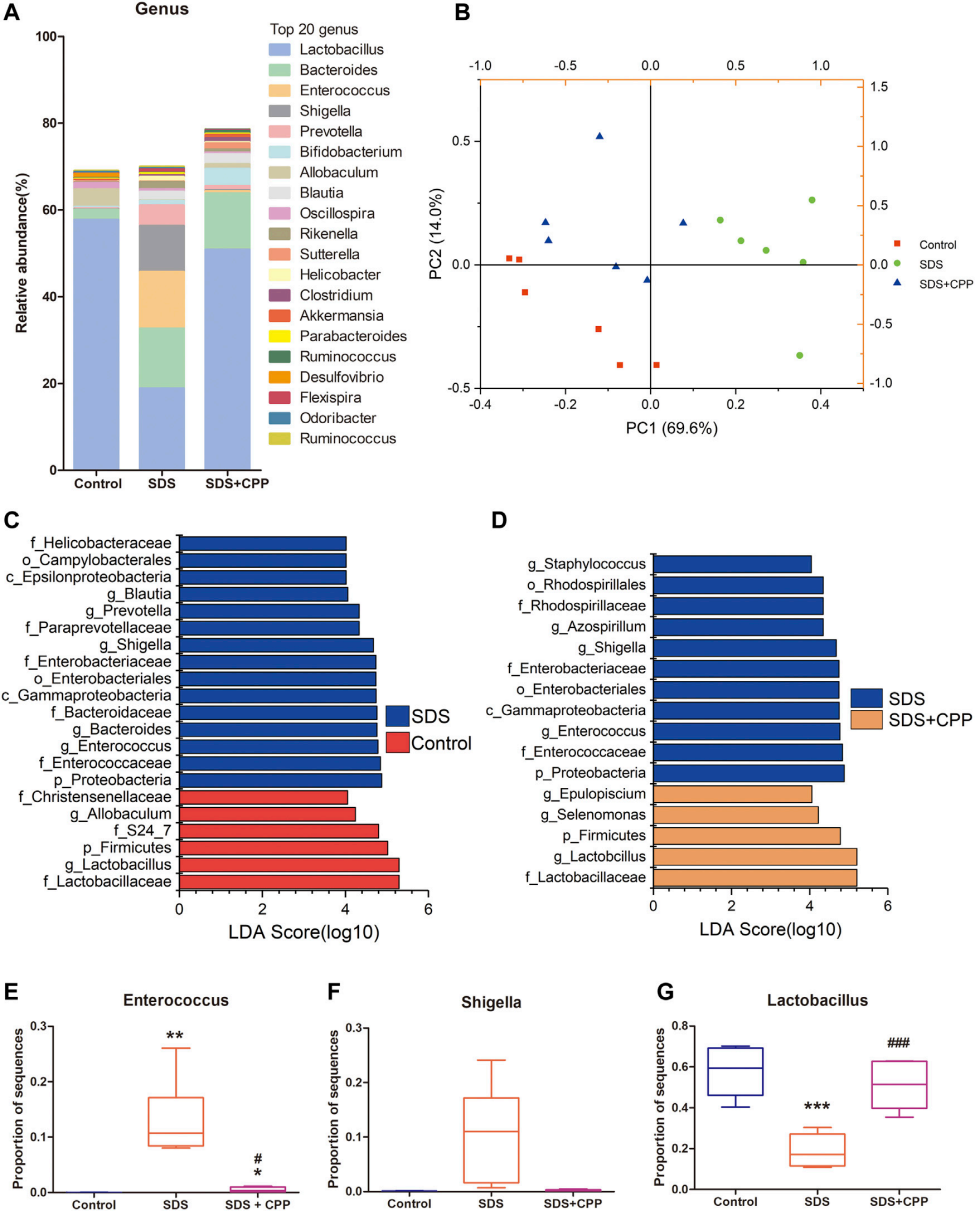
Effect of CPP gavage on the gut microbiota. **(A)** The intestinal microbiota composition analysis at genus level, the top 20 genera in control, SDS and SDS + CPP groups were visualized by histogram. **(B)** PCA analysis of gut microbiota based on the OUT data of each sample in the three groups. **(C)** LEfSe analysis of the dominant biomarker taxa between the control and SDS group. **(D)** Discriminative taxa determined by LEfSe between SDS and SDS + CPP group (log 10 LDA > 4.0). Relative abundance of **(E)** *Enterococcus*, **(F)** *Shigella*, and **(G)** *Lactobacillus* in colonic contents of the three groups. Data were expressed as box-whisker plots **(E–G)**. The boxes indicate the 25th to 75th percentiles of data, and lines within the box represent median values. The whiskers represent the lowest and highest value (*n* = 6). *p < 0.05, **p < 0.01, ***p < 0.001 *vs.* mice in control group; ^#^ *p* < 0.05, ^##^ *p* < 0.01, ^###^ *p* < 0.001 *vs.* mice in SDS group. LDA, linear discriminant analysis.

High-throughput sequencing of the 16S rRNA gene was performed to determine the microbiota profiles of the 18 colonic content samples from mice. A total of 697,386 high-quality sequences were obtained after eliminating low-quality and chimeric sequences (Supplementary Table S2). Eventually, 21,989 ASVs were obtained using QIIME2. There were 5,538 unique ASVs in the control group, 4,461 in the SDS group, and 4,446 in the SDS + CPP group (Supplementary Figure S3). The specific analysis of α-diversity values including Simpson, Shannon, Chao 1 and Paith_pd, suggested that there was no significant difference in microbiota richness or diversity among the groups in this study (Supplementary Table S3). Figure 2A shows the relative abundances of the top twenty at genus level. *Lactobacillu*s was the predominant bacterial genus in the control group, with an abundance of 57.72%. However, in the SDS group, *Lactobacillus* (18.87%), *Bacteroides* (13.78%), *Enterococcus* (13.04%) and *Shigella* (10.61%) were the predominant bacterial genera. Notably, the bacterial genera in the SDS + CPP group were quite different from those in the SDS group. The abundances of *Lactobacillus* (50.85%) and *Bifidobacterium* (3.95%) were significantly increased, while the abundances of *Enterococcus* and *Shigella* were significantly decreased in the SDS + CPP group compared to the SDS group.

The overall structure of the intestinal microbiota that was determined by PCA indicated that each group was clearly separated (Figure 2B). Moreover, LEfSe analysis was conducted to identify significantly changed bacteria under the different treatment conditions. LEfSe results displayed 15 taxa that changed significantly in the SDS group compared with the control group (log10 LDA > 4.0), including *Blautia*, *Prevotella*, *Shigella*, *Bacteroides*, and *Enterococcus* at the genus level (Figure 2C). When comparing SDS and SDS + CPP groups, there were 11 different taxa with different abundances between them, and the SDS + CPP group featured the genera *Epulopiscium*, *Selenomonas*, and *Lactobacillus* (Figure 2D). SDS group featured the genera *Enterococcus* and *Shigella* regardless of being compared with the control group or SDS + CPP group, suggesting that the microbial abundance of the two genera showed strong effects on the model group. Meawhile, *Lactobacillus* was considerably enriched at the phylum, family, and genus levels in the control and SDS + CPP groups.

The specific genus of each group was analyzed separately. *Enterococcus* occurred significantly in the SDS group when compared with the control and SDS + CPP groups (Figure 2E). *Shigella* was enriched in the SDS group, CPP administration partially downregulated the relative abundance of *Shigella* induced by SDS (Figure 2F). The relative abundance of *Lactobacillus* was significantly higher in the control and SDS + CPP groups than in the SDS group (Figure 2G). Taken together, our results indicated that CPP could shape the gut microbiota in the SDS mouse model and that *Lactobacillus* was substantially enriched.

### Antibiotic Abolishes the Invigorating Spleen Effects of *Codonopsis pilosula* Polysaccharide

**FIGURE 3:**
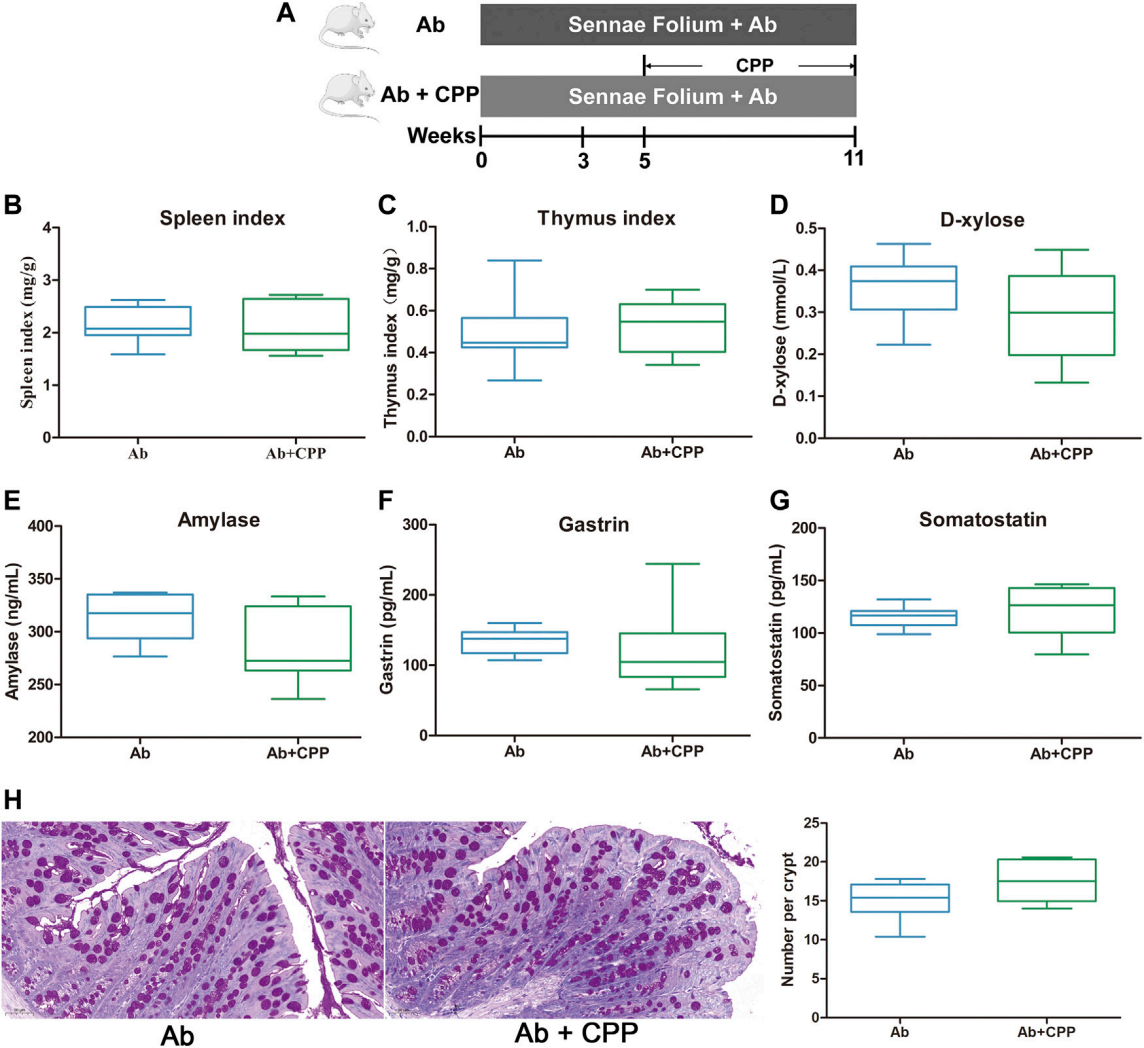
SDS remission effects of CPP depend on intestinal microbiota. Boxplots provide range, mean, minimum and maximum value. **(A)** Timeline of experimental process of mice in each group. **(B)** Effect of CPP on spleen index. **(C)** Changes in thymus index. **(D)** Serum concentration of *d* -xylose. **(E)** Serum levels of amylase reflecting digestive ability. **(F)** Serum concentration of GAS. **(G)** Serum concentration of SS. **(H)** Representative PAS-stained colonic sections (×200 magnification) in each group; and number of goblet cells per crypt in each group. Data were expressed as mean ± SEM (*n* = 8–10). Ab, antibiotic group. Ab + CPP: antibiotic + *Codonopsis pilosula* polysaccharide treatment group.

To determine whether intestinal microbiota contributes to the effects of CPP, a cocktail of antibiotics was administrated to SDS mice followed by CPP treatment (Figure 3A). CPP supplementation did not significantly increase the spleen and thymus indexes (Figures 3B,C), *D* -xylose (Figure 3D), or AMS (Figure 3E), nor did it decrease the GAS (Figure 3F) or SS (Figure 3G) serum levels. Moreover, PAS staining revealed that goblet cells were still declined after CPP treatment in Ab mice (Figure 3H). These results showed that the invigorating effect of CPP was abolished by antibiotic supplementation and that the gut microbiota was involved in the invigorating effect of CPP.

### Gut Metabolic Profiling Analysis

UPLC-Q-TOF-MS analysis was performed to assess the regulatory effects of CPP on the metabolite profile of SDS mice. A total of 221 metabolites, including 41 amino acids, 19 carbohydrates, 2 pyridines, 4 phenylpropanoic acids, 10 benzoic acids, 12 SCFAs, 47 fatty acids, 24 organic acids, 6 phenols, 28 bile acids, 15 carnitines, 5 indoles, 2 peptides, 3 benzenoids, 1 nucleotide, and 2 phenylpropanoids.

**FIGURE 4:**
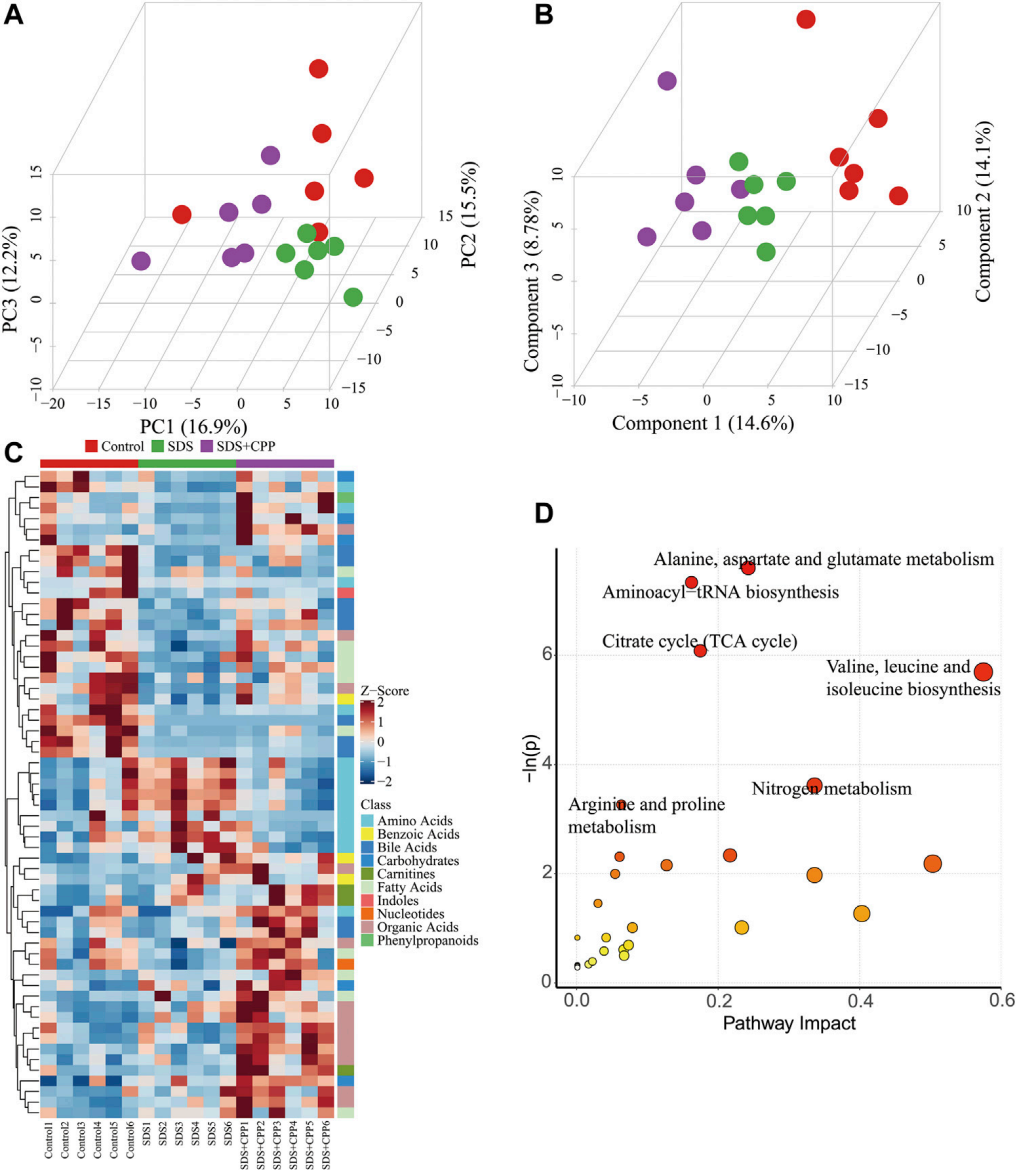
Multivariate statistical analysis, heatmap visualization of differential metabolites, and summary of metabolome pathways. **(A)** 3D PCA score plot of the metabolite profiles of control, SDS and SDS + CPP groups. Each dot represents the colonic metabolite profile of a single sample. **(B)** 3D PLS-DA score plot of different colonic content samples imply distances among groups. **(C)** Heatmap of metabolites distributions in the colonic content for each sample. Blocks in red and blue denote high and low Z-score values of metabolites, respectively. **(D)** Overview of pathway results (1) alanine, aspartate and glutamate metabolism, (2) aminoacyl-tRNA biosynthesis, (3) TCA cycle, (4) valine, leucine and isoleucine biosynthesis, (5) nitrogen metabolism, and (6) arginine and proline metabolism.

A multivariate analysis was used to reveal the clustering trends of each group, and a PCA model was established to evaluate the metabolic patterns of intestinal dysbiosis in mice (Figure 4A). Except for one plot from the control group, the samples were clearly divided into different blocks, which suggested that the SDS and SDS + CPP clusters were separated, representing the metabolic perturbation caused by the supplementation of Sennae Folium and CPP. Moreover, the supervised pattern recognition of PLS-DA showed that all three groups could be clearly discriminated (Figure 4B), indicating that each treatment generated distinct metabolite profiles.

Candidate biomarkers were identified using ANOVA and the Kruskal-Wallis test. 61 altered metabolites with a VIP > 1 and *p* < 0.05 were selected (Figure 4C). A total of 24 metabolites were increased and 37 metabolites were decreased in the SDS group compared with the control group. When comparing the SDS group with the SDS + CPP group with a fold change (FC) > 2, CPP reversed the trends in the levels of 25 metabolites: 7 amino acids, 6 organic acids, 3 fatty acids, 2 carbohydrates, 4 bile acids, 1 benzoic acid, 1 carnitine, and 1 phenylpropanoid (Supplementary Table S4). Among these, the levels of 21 metabolites were higher in the SDS + CPP group than in the SDS group. GABA and two long-chain fatty acids (Pentadecanoic acid and 9-pentadecenoic acid) were significantly higher in the SDS + CPP group than in the SDS group. In contrast, the levels of four amino acids were significantly decreased in the SDS + CPP group compared to those of the SDS group.

These differential metabolites were mapped into the KEGG pathway database, which revealed that the metabolites influenced by CPP supplementation were involved in alanine, aspartate and glutamate metabolism; aminoacyl-tRNA biosynthesis; TCA cycle; valine, leucine and isoleucine biosynthesis; nitrogen metabolism; and arginine and proline metabolism (Figure 4D). The most disturbed pathway was alanine, aspartate and glutamate metabolism.

### Gut Microbiota and Metabolites Involved in Improving Spleen Deficiency Syndrome With *Codonopsis pilosula* Polysaccharide Treatment

**FIGURE 5:**
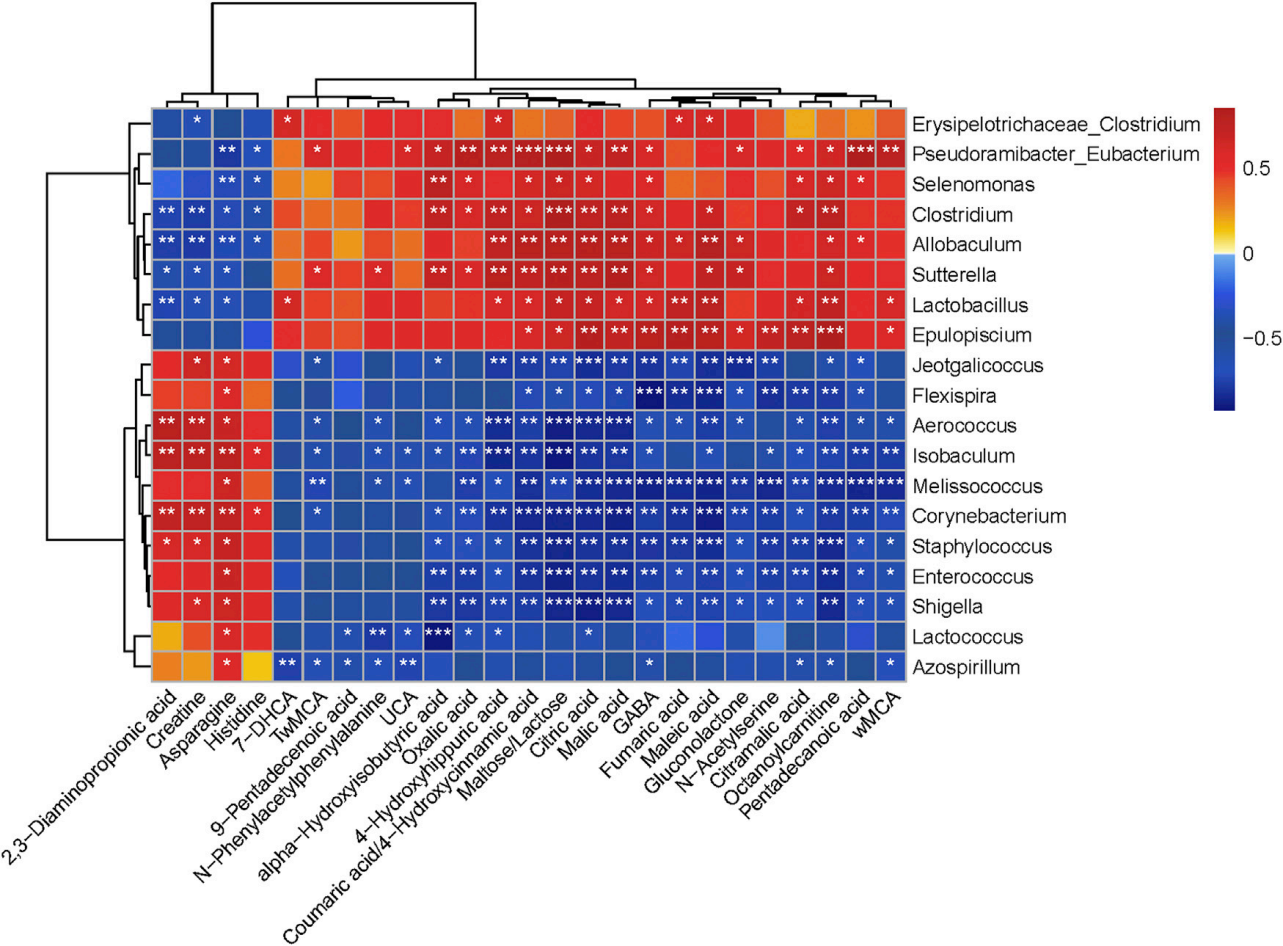
Heat map correlation between altered gut microbiota and different colonic contents metabolites, with statistical correlation values (r) > 0.7. The r value is displayed in different colors, with the red grid indicating a positive correlation and the blue grid showing a negative correlation. The larger the absolute value of r, the darker the grid color. *0.01 ˂ *p* ≤ 0.05, **0.001 ˂ *p* ≤ 0.01, ***p ≤ 0.001.

Spearman’s rank correlation analysis was performed to investigate the relationship between the effects of CPP on gut microbiota, altered metabolites, and CPP beneficial impact on SDS. The correlations between the 25 altered metabolites (FC > 2) and the 19 altered gut genera were shown in Figure 5. The genera enriched in the SDS + CPP group (*Erysipelotrichaceae_Clostridium*, *Pseudoramibacter_Eubacterium*, *Selenomonas*, *Clostridium*, *Allobaculum*, *Sutterella*, *Lactobacillus*, and *Epulopiscium*) and the SDS group (*Jeotgalicoccus*, *Fleispira*, *Aerococcus*, *Isobaculum*, *Melissococcus*, *Corynebacterium*, *Staphylococcus*, *Enterococcus*, *Shigella*, *Lactococcus*, and *Azospirillum*) behaved in the opposite manner.

Gut microbiota and altered metabolites were grouped into two clusters depending on their correlations. The first metabolite cluster contained four amino acids (2,3-diaminopropionic acid, creatine, asparagine, and histidine), and the second cluster contained 21 metabolites (6 organic acids, 3 fatty acids, 3 amino acids, 4 bile acids, 2 carbohydrates, 1 carnitine, 1 phenylpropanoid, and 1 benzoic acid). In general, there were 288 correlations (*p* < 0.05) between the metabolite types and the gut microbiota, of which *Lactobacillus* (fumaric acid, r = 0.74, *p* < 0.01; malic acid, r = 0.64, *p* < 0.05; citric acid, r = 0.71, *p* < 0.05; maleic acid, r = 0.78, *p* < 0.01; citramalic acid, r = 0.65, *p* < 0.05; octanoylcarnitine, r = 0.73, *p* < 0.01; GABA, r = 0.64, *p* < 0.05; 2,3-diaminopropionic acid, r = −0.73, *p* < 0.01; creatine, r = −0.65, *p* < 0.05; and asparagine, r = −0.66, *p* < 0.05) was found to be significantly enriched in the SDS + CPP group. Fumaric acid, malic acid and citric acid are intermediates of the TCA cycle, while amino acids and octanoylcarnitine are also related to energy metabolism. Overall, our results indicated that the abundance of *Lactobacillus* increased significantly in response to CPP treatment and related to energy metabolism substances.

The correlations between biochemical indexes and gut bacteria are shown in Supplementary Figure S4. Thirty-eight associations were identified with a *p* value ≤ 0.05 and an r value greater than 0.7. The genus *Lactobacillus* was positively correlated with AMS and spleen index, and the genera *Lactococcus*, *Enterococcus*, *Staphylococcus*, *Shigella*, *Aerococcus*, *Corynebacterium*, and *Isobaculum* were positively correlated with GAS and SS, which suggested that the changes in gastrointestinal hormones were highly related to changes in the gut microbiota of mice.

## Discussion

In this study, CPP effectively improved SDS, as mainly demonstrated by the increase of thymus index, *D* -xylose absorption, the number of goblet cells and AMS levels, and by the reduction of GAS and SS levels. Meanwhile, CPP exert invigorating spleen effect in a gut microbiota dependent manner. Our findings also revealed that CPP could significantly improve SDS induced by Sennae Folium through the regulation of the intestinal microbiota and energy related metabolisms and pathways.

Thymus, an important central immune organ where T lymphocytes develop and proliferate. Clinical studies have shown that the mRNA levels of CD9, CD164, PF4, and RARB in the peripheral blood of patients with SDS are decreased, which was involved in the decline of the immune function of patients (Li C. et al., 2017). In the present study, CPP can reverse the decrease of thymus index in spleen deficiency mice, which suggested that CPP have potential to improve immune function of SDS. In SDS, the digestive functions are affected by insufficient secretion of AMS (Xue et al., 2018), and the *D* -xylose absorption declines due to the decreased activity of Na^+^-K^+^-ATPase (Rolles and Kendall, 1972; Zhu et al., 2018). In addition, digestive function disorders can also alter the production of gastrointestinal hormones (Cui et al., 2014). After treatment for 6 weeks, CPP not only dramatically up-regulate *D* -xylose and AMS levels, but also callback gastrointestinal peptide. Thus, we concluded that CPP exert the effect of enhancing gastrointestinal absorption function in SDS.

Our previous experiment indicated that the effective dose of CPP for tonifying spleen was 1600 mg/kg (Meng et al., 2021). According to the dosage in mice experiment, human equivalent dose (129.6 mg/kg) was obtained by multiplying the Km factor 0.081 (mice Km factor) (Nair and Jacob, 2016). Therefore, an adult should take 7.78 g of CPP every day under the condition that the average adult weight is assumed to be 60 kg. This study provides a dose reference for the clinical trial of CPP in the treatment of SDS, dosage of clinical trial still need to be confirmed by further experiments. Due to the limited investigations of CPP on spleen deficiency, the present study focuses on the invigorating spleen effect of CPP on gut microbiota in mice with SDS.

An imbalance in intestinal microecology is commonly associated with the development of SDS in both clinical cases and experimental animals (Qiu et al., 2017; Lin et al., 2018a; Lin et al., 2018b; Xue et al., 2018; You et al., 2020). We found that the disordered gut microbial structure of SDS mice could be reversed in the SDS + CPP group and that the protective effect of CPP against SDS disappeared upon the application of antibiotic, which confirmed that CPP therapeutic effect on SDS depends on the regulation of intestinal microbiota.

Currently, we have identified one main component in CPP: a polysaccharide with a molecular weight of 3.78 × 10^3^ Da that is an inulin-type fructan, and its structure was confirmed as (2 → 1) linked- *D* -fructofuranose with a degree of polymerization (DP) of 31 (Li J. et al., 2017). Zou et al. (2021) also isolated two inulin-type fructans from Codonopsis Radix with DP of 19.6 and 25.2; among them, the higher DP fructan showed better prebiotic activity against lactobacilli *in vitro*. The different promoting effects on *Lactobacillus* proliferation were probably due to the higher molecular weight and DP of fructan.

*Lactobacillus* genus includes common probiotic species that can repair damaged intestinal mucosa (Wu et al., 2020), improve aerobic endurance capacity and energy harvest (Huang et al., 2018), and resist the invasion of *Shigella flexneri* in human intestinal epithelial cells (Song et al., 2020). Some studies have demonstrated that in the gut microbiota of SDS rats *Lactobacillus* decline and *Proteobacteria* increase (Ma et al., 2019; Gao et al., 2020; Li et al., 2020; You et al., 2020). Similarly, we observed that in SDS mice *Shigella* abundance increased, whereas CPP intervention markedly enriched *Lactobacillus* and inhibited *Shigella*. Thus, *Lactobacillus* may be a beneficial genus in the treatment of SDS, although the probiotic mechanism of CPP requires further research.

**FIGURE 6:**
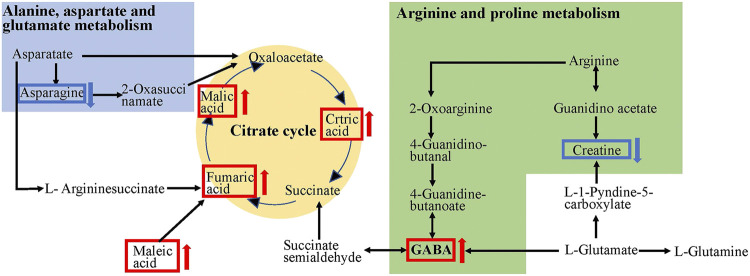
TCA cycle and amino acids metabolic pathways affected by CPP. The red arrows indicate rise and blue arrows indicate decline in the SDS + CPP group compared with the SDS group.

The metabolism of the gut microbiota has been reported to be 100 times higher than that of the liver (Possemiers et al., 2011). The metabolites produced by the gut microbiota through fermentation might be responsible for the beneficial effects of polysaccharides on the host. In this study, the concentration of 21 metabolites significantly increased in the SDS + CPP group, and the intestinal microbiota strains enriched by CPP increased the production of metabolites related to the mitochondrial TCA cycle and amino acid pathways (alanine, aspartate, and glutamate metabolism; valine, leucine, and isoleucine biosynthesis; and arginine and proline metabolism), as well as the other “energy conversion” related pathways, such as nitrogen metabolism. A previous clinical study showed that patients with spleen-yang deficiency syndrome (SYDS) have energy and carbohydrate metabolism disorders (Lin et al., 2018a). Li et al. identified 141 differentially expressed proteins between SDS rats and the control group and determined that these proteins were mainly involved in the lipid, energy, and carbohydrate pathways (Li C. et al., 2017). Our findings also agreed with a previous NMR analysis that proved that honey-fried CR affects spleen-asthenia rats by regulating glycometabolism, amino acid, lipid, and nucleotide metabolism (Hao et al., 2019). Studies have found that the Fuzi Lizhong pill can cure SYDS primarily by affecting the TCA cycle, sphingolipid metabolism, and histidine metabolism (Zhang et al., 2021). The TCA cycle is the core pathway that provides energy. Some amino acids such as alanine, aspartate, glutamate, arginine, and proline can be transformed into intermediates of the TCA cycle to provide more energy and overcome the energy deficit caused by SDS (Figure 6). Therefore, we proposed that CPP exerts a spleen-strengthening effect through the induction of mitochondrial energy metabolism.

Additionally, we observed that GABA levels increased after CPP supplementation, and the correlation analysis indicated that the abundance of *Lactobacillus* was positively correlated with the concentration of GABA in the colonic contents of the mice. *Lactobacillus* has been previously reported to be the main bacteria producing GABA (Yılmaz and Gökmen, 2020). In *L. brevis*, maltose can increase GABA production (Lim et al., 2017). Our metabolomic results revealed that the maltose concentration in the SDS + CPP group was higher than that in the SDS model group. Thus, the development of CPP enriched with probiotics and GABA (postbiotic) to relieve SDS is expected to be explored and exploited in the future.

This study established links between probiotics, microbiota-related metabolites, and biochemical parameters after CPP treatment in SDS mice. CPP administration can effectively alleviate SDS by improving the structure of intestinal microbiota and its metabolites, regulating the level of gastrointestinal hormones, promoting gastrointestinal digestion and absorption function, and improving body immunity. The structure of the intestinal microbiota changed significantly after CPP administration by increasing the *Lactobacillus* abundance in the colon. CPP mainly affected the TCA cycle and amino acid metabolism. The production of metabolites by the gut microbiota could be the mechanism implicated in the nourishing spleen effect of CPP. Further studies should be carried out to determine the relationship between TCA, GABA, and *Lactobacillus* in the pathogenesis of SDS.

## Data Availability

The datasets presented in this study can be found in online repositories. The names of the repository/repositories and accession number(s) can be found below: https://www.ncbi.nlm.nih.gov/, PRJNA796488.
