# Supplementary figures and images for: Codonopsis pilosula Polysaccharide Improved Spleen Deficiency in Mice by Modulating Gut Microbiota and Energy Related Metabolisms
Source: Front Pharmacol. 2022 Apr 26;13:862763. doi: 10.3389/fphar.2022.862763 (PMC9086242; doi:10.3389/fphar.2022.862763)

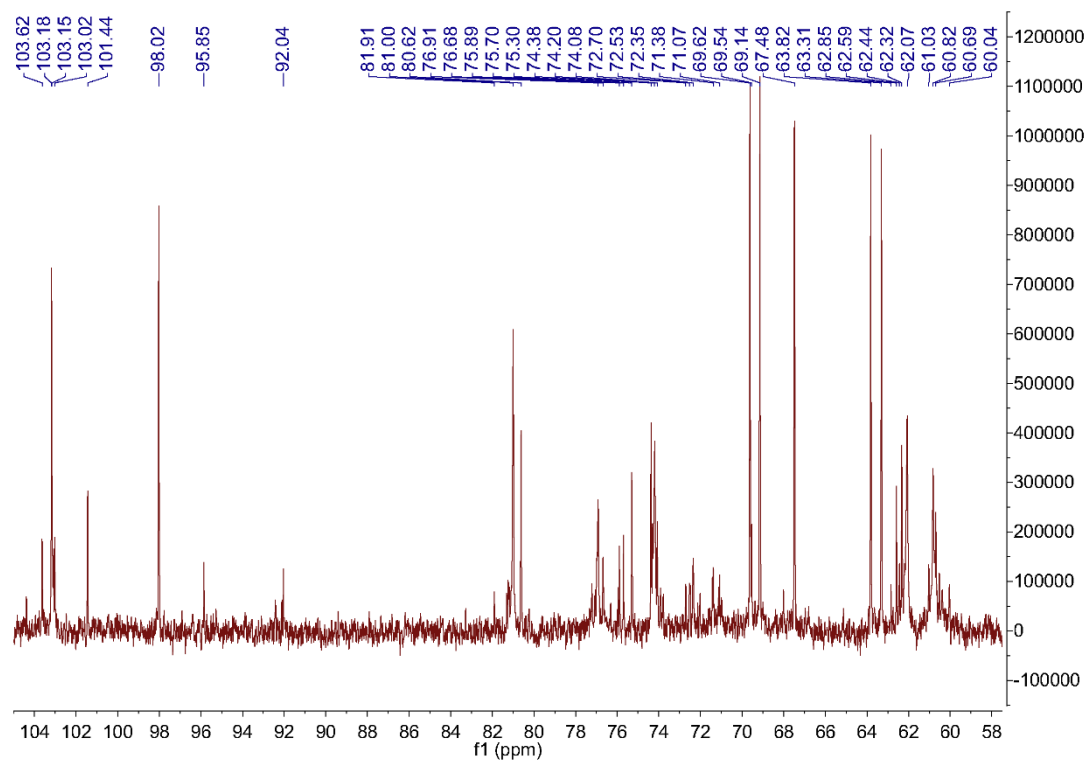

**Supplementary Figure S2**  $^{13}\text{C}$  NMR spectrum of CPP.

Supplement: Supplementary file 1 [file DataSheet2.PDF]
