# Supplementary material for: Codonopsis pilosula Polysaccharide Improved Spleen Deficiency in Mice by Modulating Gut Microbiota and Energy Related Metabolisms
Source: Front Pharmacol. 2022 Apr 26;13:862763. doi: 10.3389/fphar.2022.862763 (PMC9086242; doi:10.3389/fphar.2022.862763)
Supplement: Supplementary file 2 [file DataSheet4.PDF]

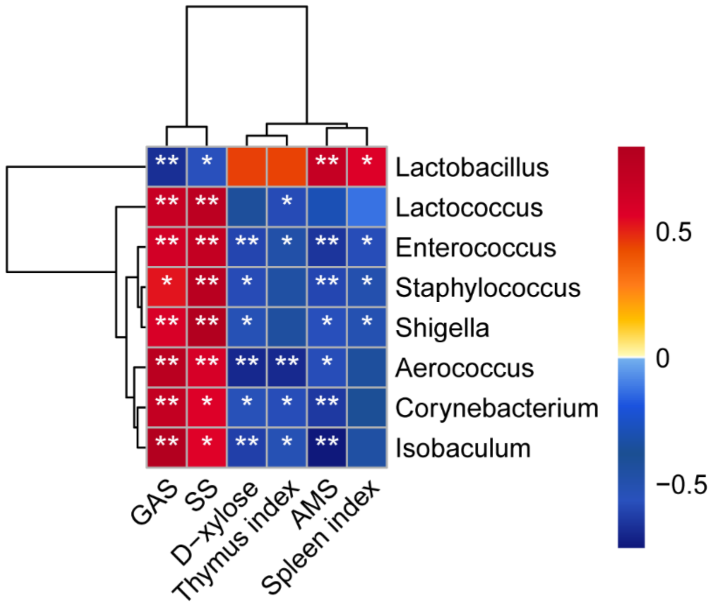

Supplementary Figure S4 Heatmaps were used to represent the relationship between flora and biochemical indexes in the Control, SDS and SDS + CPP groups. Blue grids indicate negative correlations and red grids indicate positive correlations, the absolute value of r is closely related to the depth of color.
