# Supplementary material for: Codonopsis pilosula Polysaccharide Improved Spleen Deficiency in Mice by Modulating Gut Microbiota and Energy Related Metabolisms
Source: Front Pharmacol. 2022 Apr 26;13:862763. doi: 10.3389/fphar.2022.862763 (PMC9086242; doi:10.3389/fphar.2022.862763)
Supplement: Supplementary file 3 [file Table1.DOCX]

**Supplementary Table S1** Mole ratio of monosaccharides in CPP.

| **Name** | **RT** | **Mole ratio** | **Area** |
| --- | --- | --- | --- |
| Galactosamine hydrochloride | 8.884 | 0.002 | 0.14 |
| Arabinose | 10.2 | 0.024 | 0.862 |
| Glucosamine hydrochloride | 11.284 | 0.004 | 0.312 |
| Galactose | 12.784 | 0.010 | 0.317 |
| Glucose | 14.542 | 0.342 | 6.029 |
| Fructose | 19.775 | 0.618 | 1.978 |
