# Supplementary material for: Codonopsis pilosula Polysaccharide Improved Spleen Deficiency in Mice by Modulating Gut Microbiota and Energy Related Metabolisms
Source: Front Pharmacol. 2022 Apr 26;13:862763. doi: 10.3389/fphar.2022.862763 (PMC9086242; doi:10.3389/fphar.2022.862763)
Supplement: Supplementary file 4 [file DataSheet3.PDF]

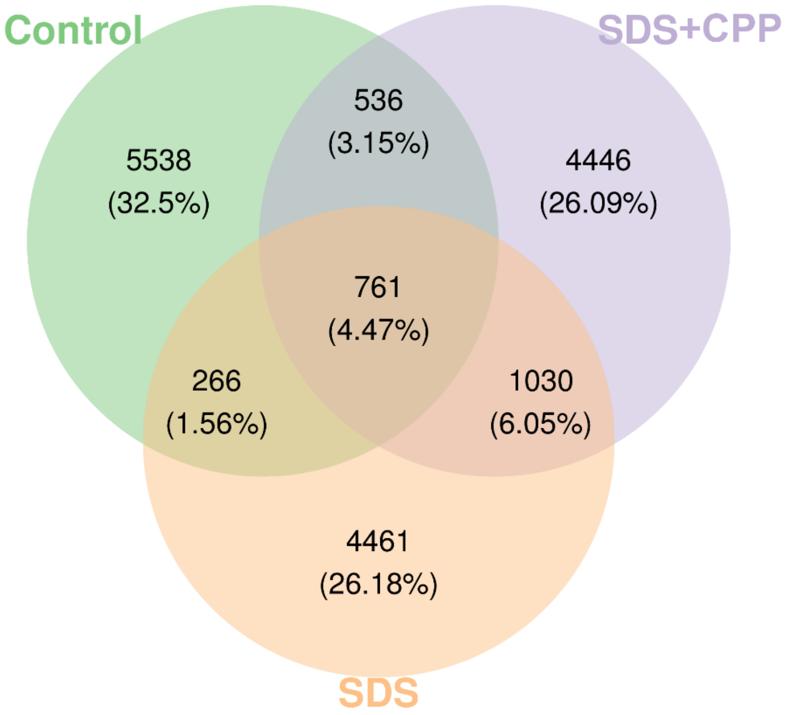

Supplementary Figure S3 Venn diagram showing the similarities and differences between groups based on OTUs. It is showed that only 761 (4.47%) OTUs are shared by three groups. Parts with nonoverlapping represent the number of OTUs unique in the group. SDS: spleen deficiency syndrome group. SDS + CPP: spleen deficiency syndrome + *Codonopsis pilosula* polysaccharide treatment group. (n = 6).
