# Supplementary material for: Codonopsis pilosula Polysaccharide Improved Spleen Deficiency in Mice by Modulating Gut Microbiota and Energy Related Metabolisms
Source: Front Pharmacol. 2022 Apr 26;13:862763. doi: 10.3389/fphar.2022.862763 (PMC9086242; doi:10.3389/fphar.2022.862763)
Supplement: Supplementary file 5 [file Table2.DOCX]

**Supplementary Table S2** The 16S rRNA gene sequencing data of samples.

| **Sample ID** | **Input** | **Filtered** | **Denoised** | **Merged** | **Non-chimeric** | **Non-singleton** |
| --- | --- | --- | --- | --- | --- | --- |
| Control1 | 66950 | 55628 | 54360 | 50418 | 34217 | 33533 |
| Control2 | 63934 | 54001 | 52318 | 45497 | 34146 | 33490 |
| Control3 | 69214 | 57935 | 56157 | 49970 | 34986 | 33662 |
| Control4 | 66022 | 54244 | 53168 | 49857 | 33126 | 32250 |
| Control5 | 97799 | 83344 | 81078 | 72807 | 49082 | 47863 |
| Control6 | 73041 | 61878 | 60699 | 57551 | 42601 | 42107 |
| SDS1 | 65452 | 55785 | 54690 | 51235 | 33415 | 32853 |
| SDS2 | 64861 | 56195 | 54833 | 50130 | 32868 | 31890 |
| SDS3 | 64014 | 55137 | 53851 | 49871 | 31450 | 30822 |
| SDS4 | 83612 | 72082 | 68924 | 57502 | 40976 | 39575 |
| SDS5 | 79356 | 68646 | 67186 | 62390 | 40406 | 39795 |
| SDS6 | 67681 | 58358 | 57132 | 53628 | 35015 | 34551 |
| SDS+CPP1 | 72288 | 62454 | 60989 | 56056 | 36792 | 35526 |
| SDS+CPP2 | 83424 | 70912 | 69317 | 64316 | 46172 | 45431 |
| SDS+CPP3 | 75225 | 65995 | 64415 | 59696 | 39032 | 38067 |
| SDS+CPP4 | 88055 | 74522 | 73096 | 67393 | 45211 | 44270 |
| SDS+CPP5 | 84517 | 71303 | 69952 | 65725 | 41061 | 40457 |
| SDS+CPP6 | 90508 | 76364 | 75048 | 70694 | 46830 | 46186 |
| Total | 1355953 | 1154783 | 1127213 | 1034736 | 697386 | 682328 |
