# Supplementary material for: Codonopsis pilosula Polysaccharide Improved Spleen Deficiency in Mice by Modulating Gut Microbiota and Energy Related Metabolisms
Source: Front Pharmacol. 2022 Apr 26;13:862763. doi: 10.3389/fphar.2022.862763 (PMC9086242; doi:10.3389/fphar.2022.862763)
Supplement: Supplementary file 6 [file DataSheet1.PDF]

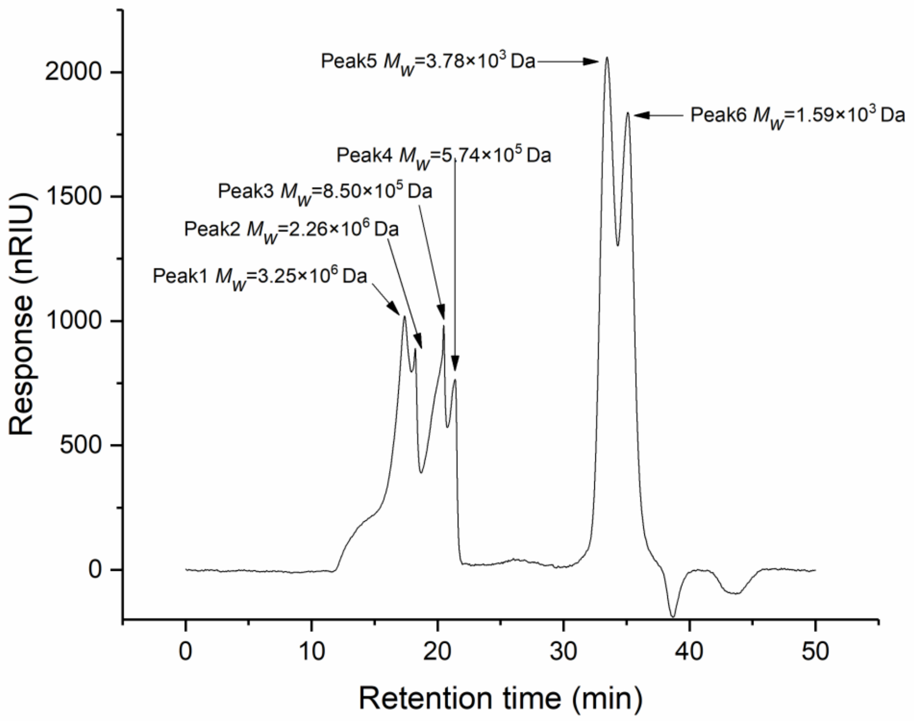

Supplementary Figure S1 HPGPC profiles of CPP. The molecular weight (M_w) distribution of Peak1 to 6 were 3.25×10^6 Da (Rt: 17.388min), 2.26×10^6 Da (Rt: 18.230min), 8.50×10^5 Da (20.486min), 5.74×10^5 Da (21.394min), 3.78×10^3 Da (33.449min) and 1.59×10^3 Da (35.102min), respectively.
