# Supplementary material for: Codonopsis pilosula Polysaccharide Improved Spleen Deficiency in Mice by Modulating Gut Microbiota and Energy Related Metabolisms
Source: Front Pharmacol. 2022 Apr 26;13:862763. doi: 10.3389/fphar.2022.862763 (PMC9086242; doi:10.3389/fphar.2022.862763)
Supplement: Supplementary file 7 [file Table3.DOCX]

**Supplementary Table S3** The Dunn Kruskal-Wallis test for alpha-diversity index comparisons among different groups.

| **Groups** | **Simpson** | **Shannon** | **Chao1** | **Observed_species** |
| --- | --- | --- | --- | --- |
| Control | 0.954±0.012 | 7.38±0.29 | 2153.87±183.83 | 1869.35±159.14 |
| SDS | 0.97±0.005 | 7.62±0.23 | 1937.51±193.52 | 1761.95±168.52 |
| SDS+CPP | 0.97±0.004 | 7.63±0.17 | 2128.15±124.21 | 1816.55±104.91 |

The multiple comparisons showed that no significance is found between groups. SDS: spleen deficiency syndrome group. SDS + CPP: spleen deficiency syndrome + *Codonopsis pilosula* polysaccharide treatment group.
