# Supplementary material for: Codonopsis pilosula Polysaccharide Improved Spleen Deficiency in Mice by Modulating Gut Microbiota and Energy Related Metabolisms
Source: Front Pharmacol. 2022 Apr 26;13:862763. doi: 10.3389/fphar.2022.862763 (PMC9086242; doi:10.3389/fphar.2022.862763)
Supplement: Supplementary file 8 [file Table4.DOCX]

**Supplementary Table S4** Statistical analysis of differential metabolites to distinguish SDS and SDS + CPP groups.

| **No.** | **Class** | **Metabolites** | **SDS+CPP vs. SDS group** | **log_2_FC** | **HMDB** |
| --- | --- | --- | --- | --- | --- |
| 1 | Amino Acids | 2,3-Diaminopropionic acid | ↓# | -1.274 | HMDB0002006 |
| 2 | Amino Acids | Histidine | ↓# | -1.025 | HMDB0000177 |
| 3 | Amino Acids | Creatine | ↓# | -2.227 | HMDB0000064 |
| 4 | Amino Acids | Asparagine | ↓## | -2.750 | HMDB0000168 |
| 5 | Amino Acids | GABA | ↑## | 2.280 | HMDB0000112 |
| 6 | Amino Acids | N-Phenylacetylphenylalanine | ↑## | 1.193 | HMDB0002372 |
| 7 | Amino Acids | N-Acetylserine | ↑# | 1.197 | HMDB0002931 |
| 8 | Organic Acids | Malic acid | ↑## | 1.815 | HMDB0000156 |
| 9 | Organic Acids | Fumaric acid | ↑# | 1.423 | HMDB0000134 |
| 10 | Organic Acids | Citric acid | ↑### | 1.185 | HMDB0000094 |
| 11 | Organic Acids | alpha-Hydroxyisobutyric acid | ↑## | 1.262 | HMDB0000729 |
| 12 | Organic Acids | Oxalic acid | ↑## | 1.205 | HMDB0002329 |
| 13 | Organic Acids | Maleic acid | ↑## | 1.235 | HMDB0000176 |
| 14 | Fatty Acids | Pentadecanoic acid | ↑## | 1.517 | HMDB0000826 |
| 15 | Fatty Acids | 9-Pentadecenoic acid | ↑# | 1.301 | HMDB0029765 |
| 16 | Fatty Acids | Citramalic acid | ↑# | 1.517 | HMDB0000426 |
| 17 | Carbohydrates | Gluconolactone | ↑# | 2.875 | HMDB0000150 |
| 18 | Carbohydrates | Maltose/Lactose | ↑## | 3.950 | NA |
| 19 | Bile Acids | 7-DHCA | ↑# | 1.696 | NA |
| 20 | Bile Acids | wMCA | ↑## | 2.009 | HMDB0000364 |
| 21 | Bile Acids | UCA | ↑# | 1.548 | HMDB0000917 |
| 22 | Bile Acids | TwMCA | ↑# | 1.173 | NA |
| 23 | Benzoic Acids | 4-Hydroxyhippuric acid | ↑## | 1.068 | HMDB0013678 |
| 24 | Carnitines | Octanoylcarnitine | ↑## | 2.554 | HMDB0000791 |
| 25 | Phenylpropanoids | Coumaric acid/4-Hydroxycinnamic acid | ↑## | 2.010 | NA |

‘↑’ and ‘↓’denote trend of increase and decrease. #*P*＜0.05, ##*P*＜0.01, ###*P*＜0.001, respectively.
